# Postoperative results of the in situ fenestrated open stent technique for acute aortic dissection type A

**DOI:** 10.1007/s11748-022-01878-4

**Published:** 2022-10-18

**Authors:** Shuhei Azuma, Ryo Shimada, Yoshikazu Motohashi, Yasuyoshi Yoshii

**Affiliations:** 1 Department of Cardiovascular Surgery, Kyoto Katsura Hospital, Nishikyo-ku Yamadahirao-cho 17, Kyoto City, 〒615-8256 Japan; 2 Department of Cardiovascular Surgery, Shiroyama Hospital, Osaka, Japan; 3 Department of Thoracic Surgery, Hirakata City Hospital, Osaka, Japan

**Keywords:** In situ fenestrated open stent technique, Acute aortic dissection, Frozen elephant trunk

## Abstract

**Objective:**

Total arch replacement is commonly used for acute aortic dissection type A at some facilities, especially since open stent grafting became commercially available in Japan. Left subclavian artery (LSCA) reconstruction involves deep view manipulation, is difficult to expose and anastomose, and involves the risk of complications and surrounding vascular injury.

**Methods:**

We evaluated 137 patients (mean age 73.8 ± 15.6 years) who underwent total arch replacement for acute aortic dissection type A, at our hospital between September 2014 and March 2022, and divided them into two groups: 70 patients for total arch replacement with fenestrated open stent technique (FeneOS), and 67 for conventional total arch replacement with the reconstruction of three-branch cerebral vessels. We performed FeneOS by deploying the graft from the entry of the left subclavian artery into the descending aorta and fenestrating the LSCA side of the stenting portion. The four-branched artificial vessel was then anastomosed between the left common carotid artery and LSCA.

**Results:**

The surgical results of FeneOS were satisfactory and enabled significant reductions in operative time, selective cerebral perfusion time, cardiopulmonary bypass time, and lower body circulatory arrest time. Long-term observation (mean follow-up = 5.5 years) showed no left recurrent laryngeal nerve palsy or postoperative problems with left subclavian artery blood flow.

**Conclusions:**

FeneOS can minimize LSCA exposure because there is no need for LSCA reconstruction, reducing operation time and avoiding the risk of left recurrent laryngeal nerve injury and bleeding problems associated with LSCA exposure and anastomosis during left subclavian artery exposure.

## Introduction

**Fig. 1:**
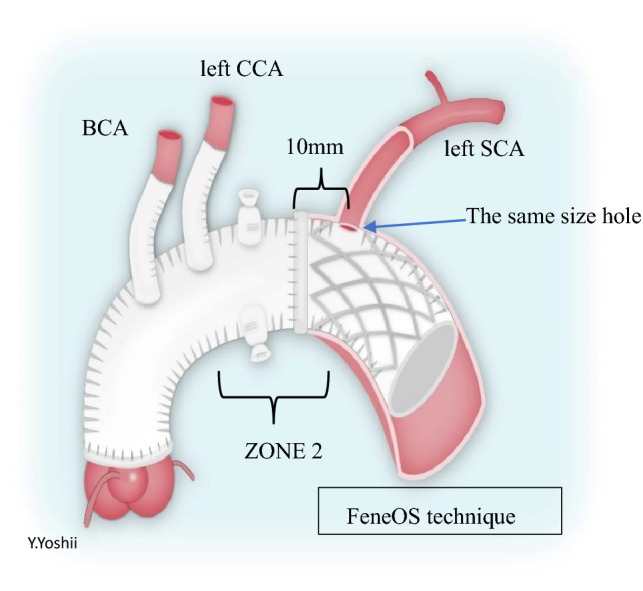
The schematic illustration showed the surgeon-made in situ fenestrated open stent technique (FeneOS).The diameter of the stent graft was selected by one of 100–110% the size of the true lumen, or the same size of the diameter of the descending aorta, determined by preoperative CT measurements. The length of the FET was selected to ensure that the distal end was placed above the aortic valve level of descending aorta. Subsequently, a hole of the same size as the entrance of the LSCA was manually created with a sharp-bladed scalpel at 10 mm from the proximal end of the FET. We then performed distal anastomosis at Zone 2 without any additional fixation procedures for the fenestration

Surgery for acute aortic dissection type A [AAD(A)] remains a challenging procedure. Total arch replacement (TAR) has been the basic technique for AAD(A). In TAR, left subclavian artery (LSCA) reconstruction often involves deep view manipulation, is difficult to expose and anastomose, and involves the risk of complications, especially left recurrent laryngeal nerve palsy [1] and vascular injury. Moreover, this procedure is time-consuming. Since open stent grafting became available in Japan, the frozen elephant trunk technique (FET) has been regularly used [2, 3]. In TAR combined with FET, we originally used the procedure of in situ fenestrated open stent technique (FeneOS) in order to simplify the operation and avoid the risk of complications. In FeneOS, a fenestration is made in the stenting portion of the open stent graft to match the entrance of the LSCA, and the distal anastomosis is performed in Zone 2 (Fig. 1). FeneOS allows minimal exposure of the LSCA because there is no need for reconstruction of the LSCA, shortening the time required for exposure and avoiding the risk of damage to the left recurrent nerve and the bleeding problems associated with LSCA exposure and anastomosis. In this study, we investigated the efficacy of FeneOS and evaluated its long-term results.

## Methods

We evaluated 137 patients [mean age 74.8 ± 16.6 years; *n* (males) = 61] who underwent TAR for AAD(A), at our hospital between September 2014 and March 2022, and divided them into two groups: TAR with FeneOS technique (FeneOS group) and conventional TAR with the reconstruction of three-branch cerebral vessels (non-FeneOS group). Frozen elephant trunk was used in the non-FeneOS group. Patients with dissections extending into the LSCA, with a primary entry very close to LSCA, or with an LSCA originating from the aneurysm were treated with conventional three- branch cerebrovascular reconstruction. FeneOS was not performed in these cases.

All patients were approached through a median sternotomy under general anesthesia in the supine position. After establishing cardiopulmonary bypass circulation, the ascending aorta was clamped, and cooling was started after cardiac arrest, with a target rectal temperature of 28–30 °C. Proximal anastomosis was performed during cooling using a four-branch vascular prosthesis. After completion of the proximal anastomosis, circulation was stopped (rectal temperature 28–30 °C), and the aortic arch was transected under antegrade selective cerebral perfusion and a commercially available FET (J Graft FROZENIX, Japan Lifeline, Tokyo, Japan) was inserted into the true lumen of the descending aorta. The diameter of the stent graft was selected by one of two methods: 100–110% the size of the true lumen, or the same size of the diameter of the descending aorta, determined by preoperative CT measurements. The length of the FET was selected to ensure that the distal end was placed above the aortic valve level of descending aorta. Subsequently, a hole of the same size as the entrance of the LSCA was manually created with a sharp-bladed scalpel at 10 mm from the proximal end of the FET. We then performed distal anastomosis at Zone 2 without any additional fixation procedures for the fenestration. The outer membrane side was reinforced with felt, and continuous sutures were made with 2–0 nonabsorbable braid sutures to form the transection. After the distal anastomosis, blood was pumped from the lateral branch of the artificial vessel to resume circulation. The reconstruction of the left common carotid and brachiocephalic arteries was performed during rewarming. All patients underwent postoperative aortic contrast computed tomography (CT) and ultrasound evaluation to evaluate blood flow in the LSCA. After discharge, outpatient follow-up was continued and patients were routinely checked for blood pressure in both the left and right upper extremities, the LSCA was examined by ultrasound, and the presence of ischemic symptoms in the left upper extremity was determined. Simple CT or contrast CT is also performed at the same time. Statistical analysis was performed using StatView (SAS Institute Inc, Cary, NC, v.26). The Kolmogorov–Smirnov test was used to determine the normality of continuous variables and *t* tests assessed differences between the two groups. *p* < 0.01 was considered significant. The present study was approved by the Institutional Review Board of Kyoto Katsura Hospital (IRB number 848).

### Early results

The preoperative background characteristics of TAR patients in the FeneOS group (*n* = 70) and non-FeneOS group (*n* = 67) are shown in Table 1. No significant differences in preoperative characteristics were observed between the two groups. Early results and intraoperative data are shown in Table 2. Early results were satisfactory in both groups, with no significant difference in operative mortality (5.7% versus 6.0% in the FeneOS and non-FeneOS groups). The FeneOS group had a significantly shorter operative time, selective cerebral perfusion time, cardiopulmonary bypass time, and lower body circulatory arrest time (*p* < 0.01). Regarding postoperative complications, no cases of left recurrent nerve palsy or postoperative problems with LSCA blood flow were observed in the FeneOS group (Table 2). On the other hand, 3 patients in non-FeneOS group had recurrent nerve palsy postoperatively and are still experiencing hoarseness and aspiration. No endoleak around the fenestration was observed.

**Table 1:** Preoperative characteristics of overall patients

Characteristics	FeneOS	Non-FeneOS	*p* value
Number of patients	70	67	–
Sex, men/women	29/41	25/42	0.89
Age, years, mean ± SD	75.5 ± 14.5	72.5 ± 10.7	0.50
Age more than 80 years old	25 (35.7%)	28 (41.8%)	0.30
Cardiac tamponade	20 (20.6%)	22 (32.8%)	0.45
Cardiopulmonary resuscitation		1 (1.5%)	0.89
Diabetes mellitus	7 (10.0%)	8 (11.9%)	0.97
Hypertension	51 (72.9%)	48 (71.6%)	0.68
Chronic obstructive pulmonary disease	8 (11.4%)	10 (14.9%)	0.56
Acute cerebral infarction	9 (12.4%)	8 (11.9%)	0.65
Old cerebral infarction	10 (14.3%)	10 (14.9%)	0.80
Ischemic heart disease	6 (8.6%)	5 (7.5%)	0.89
Marfan syndrome	0	0	1.00
Cannulation sites for the cardiopulmonary bypass			
Ascending aorta	28(40.0%)	30(44.8%)	0.66
Axillary artery	39(55.7%)	35(52.2%)	0.50
Femoral artery	3(4.3%)	2(3.0%)	0.97
Transventricular	0	0	1.00

**Table 2:** Operative data and postoperative outcomes of overall patients

Characteristics	FeneOS	Non-FeneOS	*p* value
Number of patients	70	67	–
Operative mortality within 30 days	4(5.7%)	4(6.0%)	0.83
Hospital mortality	4(5.7%)	4(6.0%)	0.96
Concomitant surgery	8(11.4%)	7(10.4%)	0.74
(Bentall/AVR/CABG)	(1/5/2)	(1/3/3)	
Operative time, minutes (except concomitant surgery)	365 ± 34.5	420 ± 62.1	*p* < 0.01
Cardiopulmonary bypass time, minutes (except concomitant surgery)	220 ± 52.7	280 ± 35.5	*p* < 0.01
Cardiac arrest time, minutes (except concomitant surgery)	135.4 ± 33.5	148 ± 40.1	0.06
Selective cerebral perfusion time, minutes (except concomitant surgery)	99.5 ± 30.4	140 ± 33.1	*p* < 0.01
Lower body circulatory arrest time, minutes (except concomitant surgery)	60.7 ± 18.6	81.5 ± 13.2	*p* < 0.01
Minimum rectal temperature, ℃	27.8 ± 4.5	27.5 ± 5.2	0.79
Reexploration for bleeding	0	1 (1.5%)	0.65
Deep site infection	1 (1.4%)	1 (1.5%)	0.62
Surgical site infection	1 (1.4%)	2 (3.0%)	0.61
Renal dysfunction, (serum creatinine > 2 mg/dl)	10 (14.3%)	11 (16.4%)	0.91
Permanent neurologic dysfunction	4 (5.7%)	4 (6.0%)	0.71
Temporary neurologic dysfunction	4 (5.7%)	3 (4.5%)	0.86
Recurrent nerve palsy	0	3 (4.5%)	0.33
Postoperative additional TEVAR	4 (5.7%)	4 (6.0%)	

In cases of stenosis of the true lumen of the descending aorta, where enlargement of the residual false lumen or large residual reentry has occurred and additional thoracic endovascular aortic repair (TEVAR) is necessary, it should be performed within 1 year after the operation. In this study, 4 patients in the FeneOS group and 4 patients in the non-FeneOS group underwent additional TEVAR with good results.

### Long-term results

**Fig. 2:**
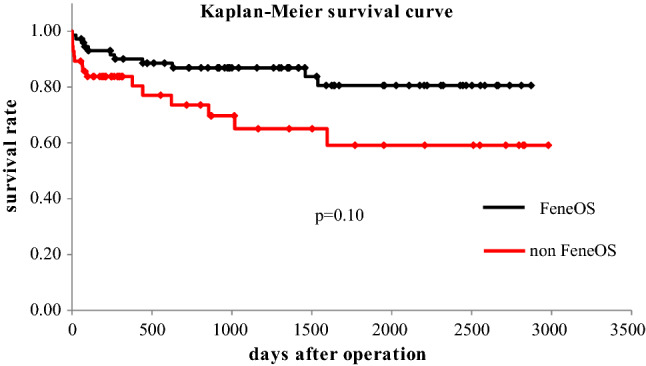
Long-term mortality (FeneOS vs. non-FeneOS)

**Fig. 3:**
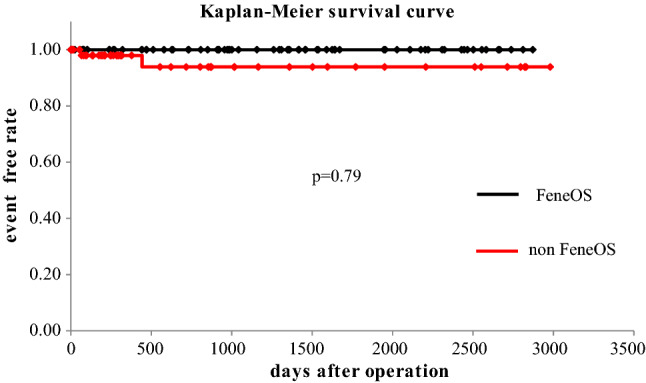
Freedom from aorta-related events (FeneOS vs. non-FeneOS)

**Fig. 4:**
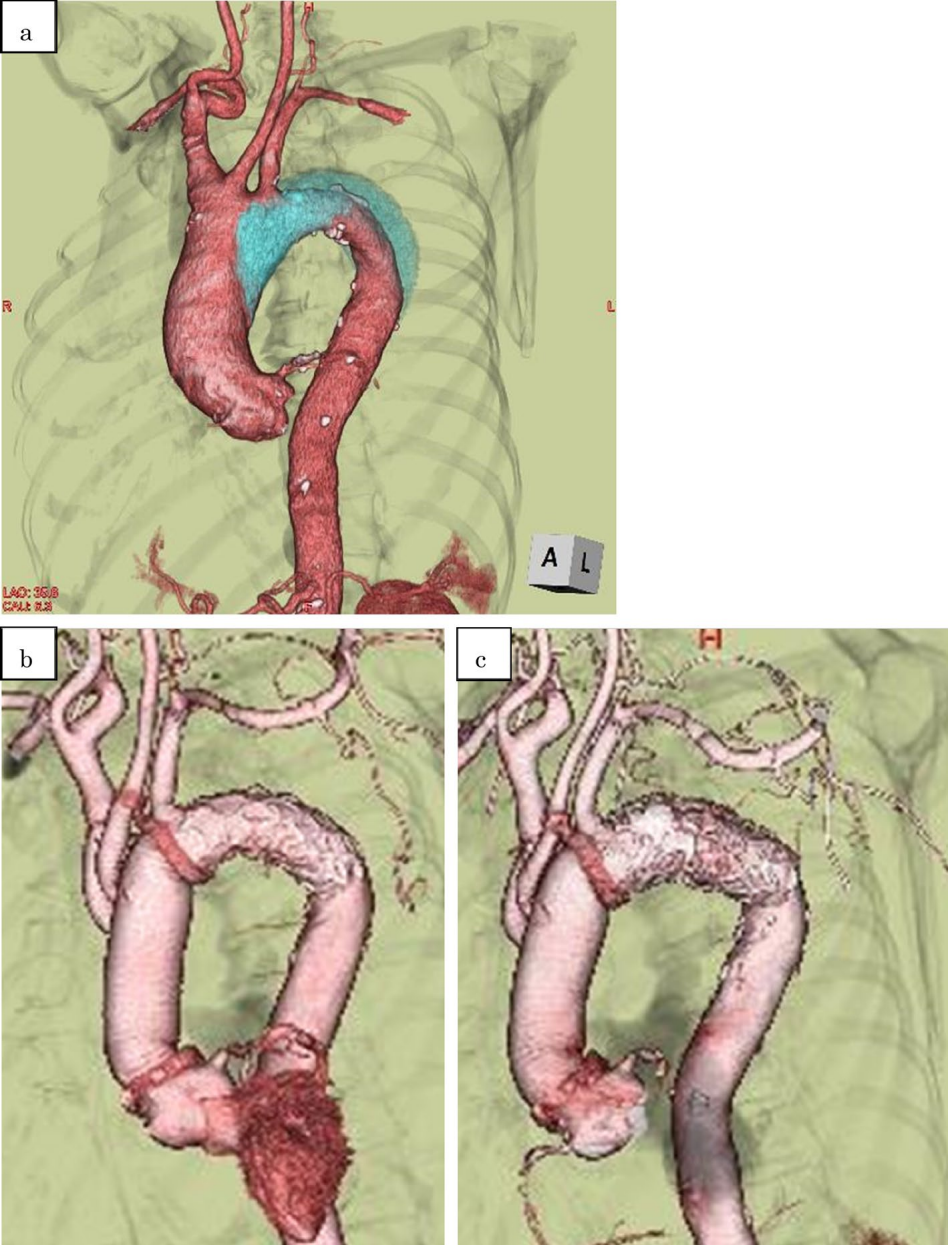
**a** Three-dimensional computed tomography (3DCT) showed acute aortic dissection type A. The blue area is the false lumen, and the SCA originates from the true lumen. **b** 3D CT 1 month postoperatively, FeneOS revealed no endoleak, and the LSCA was seen clearly through the hole of the proximal stenting portion of the open stent graft. **c** 3D CT 7.5 years postoperatively, the absence of postoperative complications, such as fenestration site occlusion or endoleak, continued throughout the long-term follow-up period (*BCA* brachiocephalic artery, *CCA* common carotid artery, *SCA* subclavian artery)

**Fig. 5:**
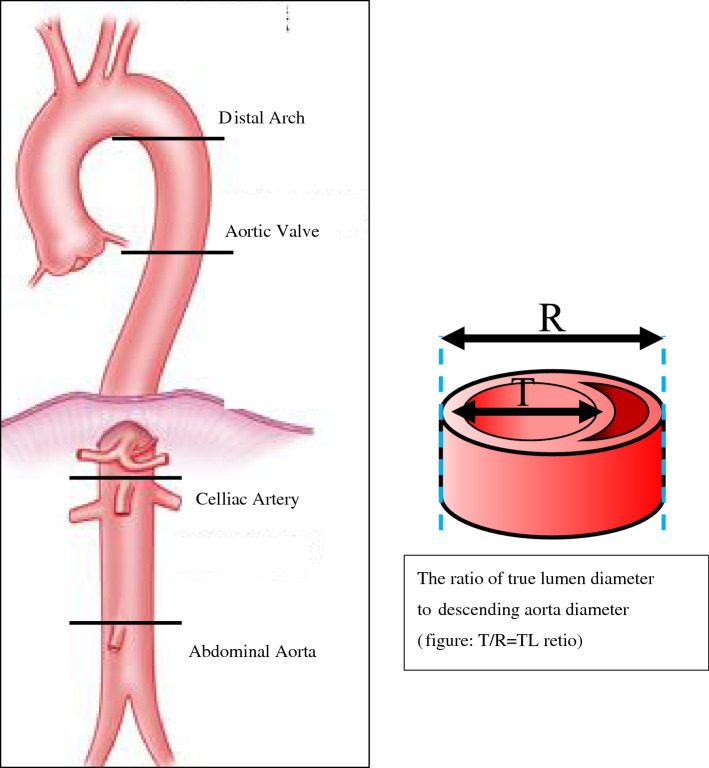

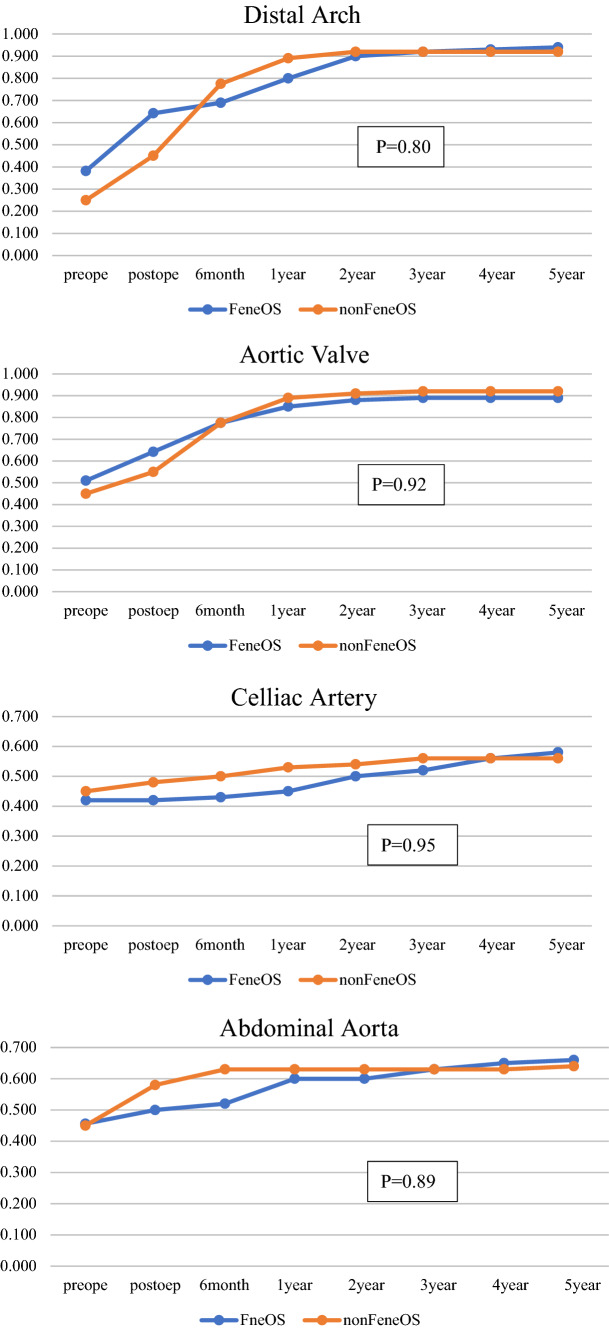
Postoperative remodeling rates in the descending aorta. Changes in the ratio of true lumen diameter to descending aorta diameter (TL ratio). The TL ratio was measured at each of 4 levels in the descending aorta (distal arch, aortic valve, celiac artery and abdominal aorta) for 5 years postoperatively. There is no significant difference between 2 groups

The long-term mortality (mean follow-up duration = 5.5 years, range 0.4–8.0 years) was satisfactory in both groups (in Fig. 2). The FeneOS group performed better than the non-FeneOS group, although the difference was not statistically significant. In the non-FeneOS group, there were 2 cases of aorta-related events: 1 patient developed distal stent graft-induced new entry 15 months postoperatively and was scheduled for TEVAR, but died suddenly before TEVAR due to rupture of the false lumen; the other patient had a rapidly enlarging false lumen in the descending aorta and died due to rupture 5 months postoperatively. In the FeneOS group, the absence of postoperative complications, such as fenestration site occlusion or endoleak-related aortic events, continued throughout the long-term follow-up period (Figs. 3, 4). Figure 5 shows there is no significant difference in the remodeling of true lumen of the descending aorta.

## Discussion

For AAD(A) in TAR patients, progress has been made towards improving acute phase prognoses and reducing false lumen diameter when open stent grafts are performed [4–6]. In the present study, we report the results of our original FeneOS procedure, in which a surgical fenestration was made in the stenting portion of the open stent graft to match the entrance of the LSCA, and a distal anastomosis was performed in Zone 2. This enabled significant reductions in operative time, selective cerebral perfusion time, cardiopulmonary bypass time, and lower body circulatory arrest time (Fig. 4).

The J-graft OPEN STENT has an internal stent structure for creating a fenestration in the stenting portion of the open stent graft, with only the stent ends fixed to the graft. It is made of thin polyester fabric, making it easy to separate the stent with a scalpel or Metzenbaum shear. Complications of FeneOS, such as endoleak and LSCA blood flow disturbance at the fenestration, were expected; however, due to the appropriate selection of the size of stent graft and creation of surgical fenestration, these complications were not observed in any of the patients, even in the long-term follow-up period. It is crucial that the size selection of the stent graft size should not exceed 110% of the diameter of the true lumen of the distal anastomosis. In addition, we believe that it is very important to create a fenestration in the appropriate location of the stent graft and that it should be equal in size, neither larger nor smaller than the LSCA entry, to prevent gutter leak.

The types of FET actually used are shown in Table 3; there were no significant differences between the two groups, but FETs in the FeneOS group tended to be longer. In both groups, the most common FET diameter was 27 mm.

**Table 3:** The length and the size of open stent graft

Characteristics	FeneOS (70 cases)	Non-FeneOS (67cases)	*p* value
The size of open stent			
21 mm	0 (0%)	2 (4.1%)	0.16
23 mm	1 (2.1%)	1 (0%)	0.31
27 mm	31 (48.9%)	29 (49.0%)	0.99
29 mm	26 (31.9%)	25 (30.6%)	0.90
31 mm	10 (12.8%)	9 (14.3%)	0.83
33 mm	1 (2.1%)	0 (0%)	0.31
35 mm	0 (0%)	1 (2.0%)	0.33
39 mm	1 (2.1%)	0 (0%)	0.31
The length of open stent			
6 cm	0 (0%)	7 (2.0%)	0.33
9 cm	30 51.1%)	36 (63.3%)	0.28
12 cm	37 (48.9%)	24 (35.8%)	0.20
15 cm	3 (4.2%)	0	

In his article, [7] Okamura has published several additional procedures that can be used to improve the results of FeneOS procedures; these are very effective and should be referred to. However, since beginning to perform the FeneOS procedure at our hospital, we have aimed for a simpler procedure and have tried to avoid exposure of the subclavian artery in most cases. The long-term results of the FeneOS, which was our primary concern, were clarified, and we found that even our simple procedure did not cause any problems.

## Conclusion

In AAD(A), FeneOS during TAR with open stent grafting was satisfactory, even throughout the long-term period. Our simple method significantly reduced the operative time, selective cerebral perfusion time, cardiopulmonary bypass time, and lower body circulatory arrest time, and no left recurrent nerve palsy was observed. FeneOS may be a useful surgical technique for TAR in AAD(A) because it reduces the time required for three-branch reconstruction and avoids risks such as left recurrent nerve palsy and the bleeding problems associated with LSCA exposure and anastomosis since FeneOS does not require exposure or reconstruction of the LSCA.
